# Therapeutics

**Published:** 1875-10

**Authors:** 


### IV. Therapeutics.

1. *Therapeutics of the Diarrhœa of Typhoid Fever.* (*Practitioner*, 1875.)

Dr. George Johnson, of London, the fecund writer on the kidney, argues in favor of an exclusively *milk* diet in the management of diarrhœa of typhoid fever. Observation teaches him that the abnormally sensitive condition of the gastro-enteric tract in this disease, is greatly aggravated by too much medicine, and too abundant alimentation. His onslaught on the mineral acid treatment, introduced a few years ago by Chambers, is especially noticeable. "I have no doubt that the comparative infrequency of the severe and obstinate diarrhœa amongst my enteric fever patients, during the last few years, is partly attributable to the *dis*continuance of this mineral acid treatment," he says. His principal dependence is careful nursing and proper feeding, and generally no medicines are necessary. The result of such a radical change in the management of typhoid fever, "has been, that diarrhœa is a less frequent symptom than formerly, and where it does occur, it is far more tractable, while tympanitic distension of the abdomen is a rare event." The sedate opium clyster and the frisky turpentine stupe, have quite disappeared from Dr. Johnson's iatrotechnic repertoire, in typhoid fever cases. His main reliance is a milk diet; milk has an "antilaxative, and even constipating effect in various morbid states, and when given alone it is one of the best antidotes for the diarrhœa of typhoid fever." In addition to milk, he gives, usually, a very small amount of beef tea and two raw eggs, every twenty-four hours. The medicines used are the "yellow mixture," which is simply colored water, "an exceptional dose of chloral to procure sleep, and a tonic during convalescence."

2. *Apocynum Cannabinum in Dropsical Affections.*

The value of this drug in dropsies was discussed at some length in a late meeting, May 18th, of the Kings County, N. Y., Medical Society. Dr. Hutchins related a case "of general anasarca, complicated with pleuritic effusion and hydro-pericardium," in a man 60 years of age, with extreme dyspnœa, from enormous distension. All diuretic means tried had failed, and at last apocynum was used. The part of the plant that is officinal is the root, but experience proves that the BARK *of the root* ONLY is efficacious. This fact was not made known to the doctor until after a few trials of some of the ordinary shop preparations of the drug, which trials were failures. Accordingly a fluid extract was made of the root's bark, and given to the patient. In forty-eight hours, "the man who had been so frightfully distended was reduced to a skeleton." The diuretic action of this agent is powerful. The amount of urine passed under its influence was said to be "incredible" in Dr. Hutchins' case. The patient lived for a year after the above named medication, and during that time "the water never accumulated, any disposition thereto being immediately relieved by the infusion of this drug."

Prof. Armour related the particulars of similar success in two cases of general anasarca. The patients were suffering from "immense dropsical accumulations, and all the usual remedies had failed." After the administration of apocynum, and within seventy-two hours, the patients were drained almost to skeletons. The professor "has but little confidence in using cathartics, diuretics, apocynum or any other remedial measure in removing fluid effusion in close cavities, when it results from acute or sub-acute *inflammation* of serous tissues." If the effusion be inflammatory *ex*udation, surgical means only will relieve the patient. If the effusion be a *trans*udation, *then* apocynum, diuretics and hydragogue cathartics will avail. He attributes his *successful* applications of apocynum to the former class of cases, and his *failures* to the latter.

Particular stress is laid on the necessity of obtaining a good drug, and the right part of it. If attention be not paid to this point the physician might as well amuse himself with cinnamon water.

3. *Mechanism of the Action of Jaborandi and a Summation of its Effects.*

A series of very elaborate articles, entitled "Physiological and Therapeutical Studies on Jaborandi," has appeared in the new *Journal de Thérapeutique*, of Paris, since Nov. 1, 1874, from the pen of M. Albert Robin, one of the medical savans of France. Extensive qualitative and quantitative analyses of every obtainable secretion and excretion of the human body have been repeatedly made while taking jaborandi. The effects of this drug on the eye, on the bodily temperature, in health and in febrile diseases, on the pulse, (extensive sphygmographic tracings accompanying the article,) are given in tedious detail, in numbers of this journal extending over a period of nine months; and in the last number are given the "mechanism of the action and a summary of the effects" of this new therapeutic agent, which are here given in full:

The active principle, *pilocarpus* (or pilocarpia, the alkaloid of jaborandi,) produces its influence by exciting while passing through, the salivary and sudoriparous glands, its elective emunctories; the activity of the latter is prodigiously exalted; it is accompanied by a "sanguinary efflux to the hyperexcited organs and by a certain degree of general stimulation of the system."

The irritation of the secreting cells and the peripheral stimulation, are transmitted to the reflex centres, by the eisodic nervous filaments and returned to the vaso-dilator and vaso-constrictor nerves which furnish to the organs of secretion the whole of the nutritious fluid exacted by their increased action.

This, it seems to us, is the physiological mechanism by which jaborandi acts to produce the effects, studied in the course of this investigation, which are:

Diaphoresis and sialorrhœa, with the elimination of rather a large proportion of urea, of the chlorides, of ptyaline, and of the carbonates.

Secondary hypercrinias, lachrymal, nasal and tracheo-bronchial; secretory irritation of glands annexed to the digestive tube, manifest, especially, when the external effects are a little complained of.

Slight augmentation of the temperature of the skin when the blood flows into this region to supply the material to the sudoriparous glands for their secretion; cold in the cutaneous envelope in consequence of the evaporation of the sweat upon its surface.

Corresponding increase in the pulse, which augments in frequency at the onset of the sweating and diminishes with the same; diminution of the intra-vascular tension, consecutive to the augmentation of capacity of the general circulatory system; irregularity of the beats of the heart when given to those with cardiac troubles, with a sort of toxic asystolia, apparently resulting from a disturbed action of the medicament upon the innervation of the heart.

Diminution of the quantity of urine secreted; but this is not in sufficient quantity to compensate for the fluids lost through the agency of the skin and salivary glands, in the same way that elimination is accomplished, although the kidneys may be liberated from a portion of their functional work.

A diminution in the phenomena of disassimilation, very trifling, as can be shown by an actual falling off of urea, which, however, is disproportionate to the energy of secretory activity, and indeed, seems independent of it; diminution of the quantity of uric acid eliminated by the urine, probably owing to a diminution in the intra-organic formation of this acid; and if it be admitted that uric acid is one of the conditions in which the albuminoid materials of the tissues are eliminated, before reaching a complete oxydation of which urea is the limit, the diminishing of this acid compared with that of urea will be still more an argument for our opinion as to the lessening of disassimilative combustion.

Diminution of the chlorides.

After the action of jaborandi, there is relatively greater energy in disassimilation, especially when the hypercrinitic effects of the medicament have been produced with great activity.

Finally, a diuretic action of jaborandi taken in fractional doses.

Such is a hasty schedule of the principal physiological effects of jaborandi.

4. *Gelsemia. Its Physiological Action.*

Dr. J. Ott, Demonstrator of Physiology in the University of Pennsylvania, after a prolonged series of experiments enumerated in the *Philadelphia Med. Times*, July 31, 1875, which he made in the laboratory of Prof. F. G. Smith, gives the following summation of his observation on the action of this alkaloid of gelseminum.

1st. In cold blooded animals it paralyzes first the sensory ganglia, and then the motor ganglia in the central nervous system. This order is reversed in warm blooded animals.

2nd. It diminishes the pulse and pressure.

3rd. This decrease of pulse rate is due to lessened irritability of the excito-motor ganglia of the heart.

4th. The fall of pressure is due to the diminution of cardiac irritability and vaso-motor tonus.

5th. It decreases the respiration through a paralyzing action on the respiratory centres.

6th. It dilates the pupil.

7th. It reduces the temperature.

5. *Chloral versus Strychnia Poisoning. Another Case.*

Dr. J. W. Winslow, of East Hampton, Mass., adds another case of successful treatment of strychnia poisoning with chloral, to the many already recorded. He writes to the *Philadelphia Med. and Surg. Reporter*, Aug. 14, 1875, "On my arrival at the house about an hour and a half after the dose was taken (half a teaspoonful of strychnia in half a tumbler of water), I was met by the father, who told me that his daughter, a young woman, had had spasms for the past hour and that they were increasing in severity—that she was "*just gone*." Cupric sulphate produced speedy emesis. "Spasms were severe and continuous." Twenty grains chloral were given, when suddenly a frightful opisthotonic convulsion occurred. "Soon as possible afterwards thirty grains more were given, when the spasms gradually diminished in frequency and force, entirely subsiding within the space of two hours." Recovery was complete.

6. *Still Another.*

The *Philadelphia Med. Times*, also bearing date Aug. 14, 1875, records another case of successful treatment of a case of strychnia poisoning by chloral, communicated by Dr. C. Bivine, of Tarrytown, Md. He gave 40 grs. of chloral when the patient—a girl of 16 years of age—had reached the stage of "general spasms, accompanied by opisthotonos and asphyxia;" 30 minutes thereafter he gave 40 grs. chloral more, and "soon after, she fell asleep." Thence to recovering he gave her chloral and potassic bromide as the case demanded. When she awoke and convulsions appeared, more chloral was given. Gradually the toxic action passed off. At the end of 48 hours there were only "slight occasional renewal of spasms and some nervous spasms." Recovery, as in the preceding case, was complete.

7. *Treatment of Neuralgia.*

Prof. von Pitha having suffered from various kinds of neuralgia for the last two years, gives an interesting account of his observations on himself, and comes to the conclusion that the hypodermic use of morphia is the only rational treatment, and generally can be employed without injurious consequences for a long period. But very small doses must be given to begin with, and gradually increased as need be. To guard against the unpleasant action of the morphine on the stomach, P. recommends the addition to the morphine of sulphate of atropia (gr. $\frac{1}{100}$) or the internal administration of muriate of quinia in black coffee.—*Wiener Med. Zeitung.*

